# Implementation of a women’s reproductive behavioral health telemedicine program: a qualitative study of barriers and facilitators in obstetric and pediatric clinics

**DOI:** 10.1186/s12884-023-05463-2

**Published:** 2023-03-11

**Authors:** Katherine R. Sterba, Emily E. Johnson, Edie Douglas, Rubin Aujla, Lisa Boyars, Ryan Kruis, Rebecca Verdin, Rachel Grater, Kathryn King, Dee Ford, Constance Guille

**Affiliations:** 1 Department of Public Health Sciences, Medical University of South Carolina, Charleston, SC 29425 USA; 2 College of Nursing, Medical University of South Carolina, Charleston, SC USA; 3 Department of Psychiatry and Behavioral Sciences, Medical University of South Carolina, Charleston, SC USA; 4 Center for Telehealth, Medical University of South Carolina, Charleston, SC USA; 5 Department of Pediatrics, Medical University of South Carolina, Charleston, SC USA; 6 Department of Pulmonary and Critical Care, Medical University of South Carolina, Charleston, SC USA; 7 Obstetrics and Gynecology, Medical University of South Carolina, Charleston, SC USA

**Keywords:** Telemedicine; perinatal mental health, Perinatal substance use disorder, Implementation science

## Abstract

**Background:**

Perinatal Mood and Anxiety Disorders and Substance Use Disorders are common and result in significant morbidities and mortality. Despite evidence-based treatment availability, multiple barriers exist to care delivery. Because telemedicine offers opportunities to overcome these barriers, the objective of this study was to characterize barriers and facilitators to implementing a mental health and substance use disorder telemedicine program in community obstetric and pediatric clinics.

**Methods:**

Interviews and site surveys were completed with practices engaged in a Women’s Reproductive Behavioral Health Telemedicine program (N = 6 sites; 18 participants) at the Medical University of South Carolina and telemedicine providers involved in care delivery (N = 4). Using a structured interview guide based on implementation science principles, we assessed program implementation experiences and perceived barriers and facilitators to implementation. A template analysis approach was used to analyze qualitative data within and across groups.

**Results:**

The primary program facilitator was service demand driven by the lack of available maternal mental health and substance use disorder services. Strong commitment to the importance of addressing these health concerns provided a foundation for successful program implementation yet practical challenges such as staffing, space, and technology support were notable barriers. Services were supported by establishing good teamwork within the clinic and with the telemedicine team.

**Conclusion:**

Capitalizing on clinics’ commitment to care for women’s needs and a high demand for mental health and substance use disorder services while also addressing resource and technology needs will facilitate telemedicine program success. Study results may have implications for potential marketing, onboarding and monitoring implementation strategies to support clinics engaging in telemedicine programs.

**Supplementary Information:**

The online version contains supplementary material available at 10.1186/s12884-023-05463-2.

## Background

Perinatal Mood and Anxiety Disorders (PMAD) and Perinatal Substance Use Disorders (PSUD) are common and carry significant morbidity and mortality for women and children. Estimates vary but approximately 16–25% of women will experience PMAD [1] during pregnancy or in the postpartum year. Also, 9–14% [2] of women will use harmful substances during pregnancy. The majority of women with PSUDs will abstain from substance use in pregnancy, but 80% will relapse postpartum, increasing their risk for overdose and death [3]. Recent data suggest that suicide and drug overdose combine to constitute the leading cause of maternal mortality during the postnatal period [4–8]. PMAD and PSUD increase these risks [5, 9, 10] and are associated with significant maternal and child morbidity [11–17].

Fortunately, treatments such as evidence-based psychotherapy and medication are effective in reducing the maternal and child morbidity and mortality associated with PMADs and PSUDs [17, 18]. However, the vast majority of women do not receive treatment due to barriers to care. Common barriers include lack of transportation or childcare necessary to obtain treatment as well as insufficient availability of specialty providers and lack of timely access to services. Furthermore, women often refuse treatment in psychiatric settings due to the stigma associated with mental illness and substance use [19, 20].

Delivering treatment for PMAD and PSUD to pregnant and postpartum women in obstetric and pediatric practices via telemedicine is one potential solution to overcoming barriers and increasing access to treatment for women. However, telemedicine service delivery can pose an additional set of implementation challenges including the need for dedicated space, equipment and staff to assist with telemedicine visits in community clinics. Using qualitative methods, the objective of this study was to characterize the barriers and facilitators to implementing a telemedicine program for the treatment of PMADs and PSUDs in obstetric and pediatric providers in the outpatient community practice setting.

## Methods

The Consolidated Criteria for Reporting Qualitative Research (COREQ) checklist was used to guide methods and reporting [21]. Qualitative methods were selected for an in-depth exploration of telemedicine experiences in the Women’s Reproductive Behavioral Health Telemedicine (WRBT) program at the Medical University of South Carolina (MUSC). The WRBT was developed at MUSC in 2016 to offer mental health and substance use disorder telemedicine services to community obstetric and pediatric practices. The WRBT Medical Director at MUSC established relationships with clinics through outreach in South Carolina. Protocols were developed for setting up programs in community clinics to deliver mental health and substance use disorder telemedicine services to community clinics’ patients. No formal readiness assessment was conducted with participants and clinics employed their own strategies for identifying and screening patients through clinical care. Referral processes were also set up between the clinics and MUSC to schedule and complete appointments. All telemedicine visits were conducted on-site at the community clinic. All patients were initially evaluated by a psychiatrist; a treatment plan was created in collaboration with the patients with evidence-based therapy such as Cognitive Behavioral Therapy or Interpersonal Psychotherapy and medication management if appropriate. In accordance with the Ryan Haight Act [22], patients requiring a prescription for a controlled substance such as buprenorphine were seen in person first per federal and state requirements for prescribing controlled substances via telemedicine.

Six practices were selected for this study based on their geographic location, lack of access to services and variability in the number of telemedicine referrals completed. Data were collected from July 2019 to February 2020. The Medical University of South Carolina’s Institutional Review Board approved this study and granted a waiver of written informed consent [Pro # 00086783]. Verbal consent was secured and participants received a $10 giftcard.

### Guiding model

The Exploration, Preparation, Implementation, Sustainment (EPIS) framework was used to guide this study [23, 24]. This implementation framework highlights a comprehensive set of factors that may influence implementation including inner context, outer context, bridging and innovation factors. In this study, potential inner context factors included influences internal to the clinic (e.g., leadership) while potential outer context factors included influences external to the clinic (e.g., professional guidelines). Finally, other influences that link inner and outer context factors (bridging factors) and characteristics of the telemedicine program itself (innovation factors) were considered.

### Recruitment and data collection

Each selected WRBT clinic’s practice manager was contacted by email and phone to discuss the study. All practices contacted were willing to enroll and the practice manager completed an online survey to assess practice characteristics (e.g., practice type, number of providers, demographic characteristics of patients) and telemedicine referrals (completed, cancelled, no-shows). Additionally, practice managers selected clinic team members currently involved in and most knowledgeable about the telemedicine program to participate in online discussion groups or interviews based on preference and availability. Online groups were conducted over Zoom with video and audio for convenience in the busy clinical setting. Finally, the MUSC clinicians and staff from the WBRT program (referred to below as the WRBT telemedicine team) completed in-person discussion groups on-site at the academic medical center.

Two female interviewers (KS, EJ) with doctoral-level training in qualitative methods moderated discussion groups using a structured interview guide informed by EPIS (Table 1). Interviewers were not involved in the telemedicine program and did not know participants. One investigator moderated discussions while the second investigator took field notes, making observations about respondent comments and body language. Each clinic had audio and video capability and all communication was verbal (i.e., no chat functions were used). Discussion groups were audio-taped and field notes were taken to inventory emerging themes and observations. Interviews lasted 20–55 min and were conducted until saturation was achieved [25, 26].

**Table 1:** Structured Interview Guide

Interview Questions
**Background**
Please describe your job at [site] and your role in the telemedicine program.
Tell us about the types of patients you serve in your clinic and the activities and initiatives that are of highest priority for quality improvement in your practice.
Tell us about the practices your clinic had in place for identifying, screening and providing mental health [and substance abuse] services for women before the telemedicine program.
**Inner Context**
Thinking broadly about the culture of your clinic (or the general ways your clinic functions and its norms), were there any concerns you had about taking on and implementing the telemedicine program? Please describe.
Describe the individuals in your clinic who have been supportive of the program and how the program is run in your practice.
What was the general level of openness and commitment to implementing the program at your clinic? Why?
Please describe the resources your practice had in place or needed to support the telemedicine program.
**Outer Context**
Several professional organizations recommend screening for depression in pregnancy and postpartum as standard of care. Please describe any challenges in completing the recommended screenings for depression.
Please describe any challenges your team faced or still faces in meeting mental health (and substance abuse) needs in your patients.
**Bridging**
Describe the relationships you have developed with staff from the telemedicine program and any challenges you have observed in accessing resources to support the telemedicine program.
Tell us about the set-up process for launching the telemedicine program at your clinic. How did that go?
**Innovation**
When starting this collaboration with the academic telemedicine program, please describe how well you felt the program fit with your clinic’s mission, work processes and practices. Why?
**General Program Feedback**
Once the program started, please describe how things went with the screening, referrals and scheduling processes.
Please comment on any challenges you have experienced in delivering the program efficiently to meet patients’ needs.
Tell us about how patients have received the program and any suggestions you have to improve their experience.
In closing, please share any suggestions you have to improve the successful delivery of the program.

Note: A parallel version was used with the telemedicine team to gather their perspectives on delivering telemedicine services to community clinics

### Data analysis

A combined inductive-deductive template analysis approach [27, 28] within N-Vivo software [29] was used with an initial codebook guided by the EPIS framework [23, 24]. Two analysts read and re-read each transcript and tested the codebook with an initial set of interviews until consensus was achieved in code definitions and interpretation. The final codebook was applied to the full set of interviews and themes were examined within and across clinic and telemedicine team perspectives.

## Results

### Participant characteristics

Five online discussion groups and 1 online interview were conducted with obstetrics (n = 4) and pediatric (n = 2) practices (N = 6 discussions with 18 overall participants; see Table 2 for number of participants in each discussion; range 1–6). Clinics had varied start dates for the WRBT program from 2016 to 2019 but all participants were currently involved in the WRBT program at the time of the focus group or interview so able to share their recent experiences. The majority of clinics (n = 4) were located in rural areas, yet varied widely with respect to staffing and patient characteristics (Table 2). Clinics commonly provided care for patients who were racially diverse (35–100% African American) and covered by Medicaid (> 50%). Telemedicine encounters and status in the previous 3 months varied by clinic (0-191) (Table 2).

**Table 2:** Participating Site Characteristics

Characteristic	Clinic 1 (n = 2)	Clinic 2 (n = 2)	Clinic 3 (n = 6)	Clinic 4 (n = 1)	Clinic 5 (n = 5)	Clinic 6 (n = 2)
Practice type	PED	PED	OB	OB	OB	OB
Number of providers						
Physicians Advanced Practice APRN Nurses Physician Assistant/Nurse Practitioner Certified Medical Assistant Midwife Sonographer Genetic Counselor Lactation Consultant Social Worker Front Desk Staff Financial Counselor	2 3 0 3 0 0 0 1 0 3 0	1 0 0 1 0 0 0 0 0 3 0	5 0 0 8 0 0 0 0 0 8 0	4 0 3 4 0 1 0 0 0 4 6	4 0 1 6 2 0 0 1 0 2 0	4 0 1 0 0 1 0 0 0 1 0
Number of patients seen annually	250	200	550	660	210	360
Insurance status (percent Medicaid patients)	50	50	55	60	75	65
Race (percent African American patients)	40	35	35	40	100	40
Clinic location ^a^	rural	rural	rural	urban	urban	rural
Telemedicine program launch	October 2018	February 2019	May 2016	July 2017	April 2019	May 2016
Number of telehealth encounters (past 3 months)						
referred completed cancelled no-show	3 2 1 0	0 0 0 0	10 8 1 1	1 0 1 0	10 6 3 1	278 191 68 19

PED = pediatric; OB = obstetrics ^a^ defined by the Health Resources and Services Administration Rural Analyzer [38]

A set of 2 in-person discussion groups were conducted with the MUSC WRBT telemedicine team; two sessions were required to allow adequate time for discussion of the telemedicine program at each participating clinic from the perspective of the team delivering the program. Participants included two Reproductive Psychiatrists who provided direct patient care, the Telemedicine Medical Director who assisted with site-set-up and a Clinical Program Coordinator who served as the site liaison to facilitate ongoing site-to-site communication and service coordination (N = 4; same participants included in each discussion group). All participants were female and the average time in the telemedicine program was 3.25 years (range 2–4).

### Barriers and facilitators to program implementation

**Fig. 1:**
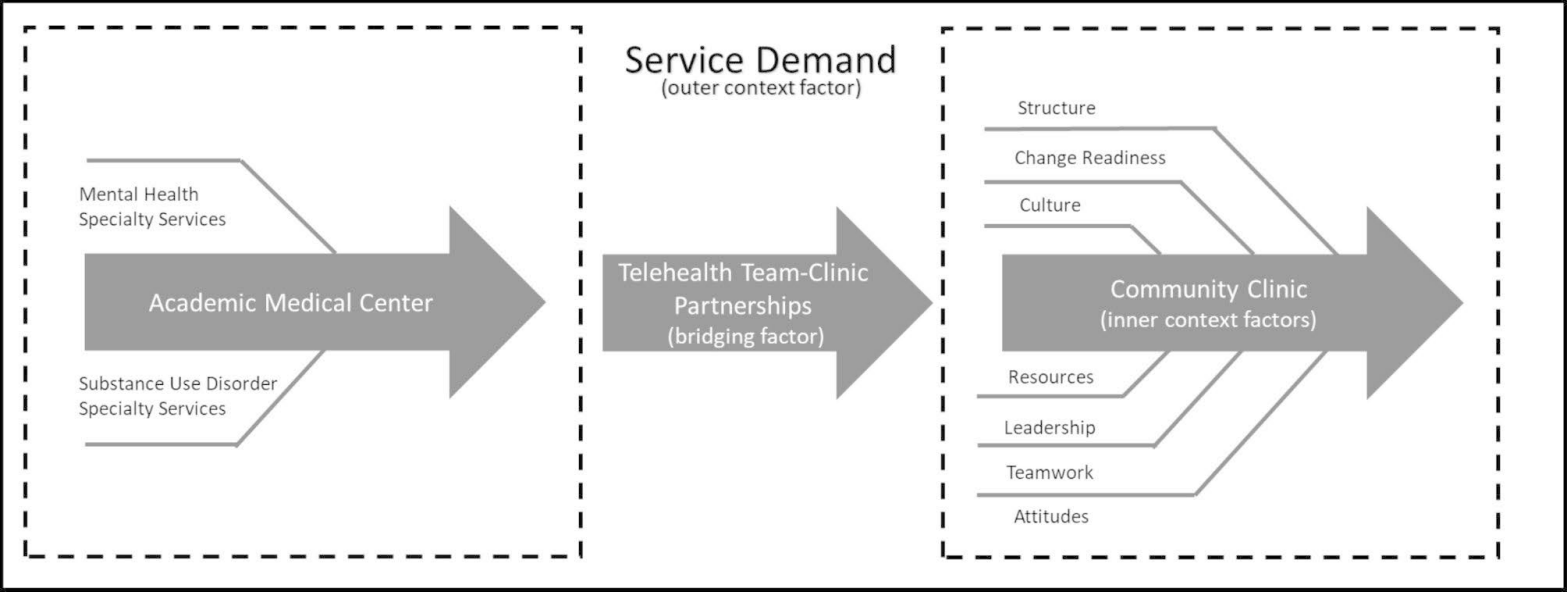
Women’s Reproductive Behavioral Health Telemedicine Implementation Process ^a^ ^a^ Adapted from the Exploration, Preparation, Implementation, Sustainment Framework [23]

As displayed in Fig. 1, unique themes were identified addressing barriers and facilitators to telemedicine service delivery. A single primary outer context factor was identified along with 7 inner context factors and one bridging factor related to the operational style of the telemedicine program. Identified themes were consistent across PMAD and PSUD care programs. Below themes are summarized and definitions and illustrative quotes are provided in Table 3.

**Table 3:** Focus Group Themes: Barriers and Facilitators to Telemedicine Program Implementation

Theme	Definition	Illustrative Quotes
**Outer Context Factor**		
Service Demand	State and regional demand, support and advocacy for maternal mental health and substance abuse healthcare needs.	*We have such a range of socioeconomics with our group that patients don’t have that access to go to [the academic medical center], so that’s huge – transportation issues are difficult for some of our patients, and so I think this is a great service to be able to offer them. (Clinic 3)* *Trying to find counseling or psychiatry for them [mothers] was near to impossible before this… And also the convenience of it being available…there was one situation where I had a mom who was really quite significantly affected, and I think that her [telehealth] appointment was like two days later, or something just ridiculously wonderful. You know? And that was something that never, there’s no way we could have gotten her to see a psychiatrist in two days without this program. (Clinic 1)* *It’s an area in our state that has some of the highest rates of opioid use and opioid overdose deaths. And so…any access to additional care for that population meant a lot to them. (Telehealth Team)*
**Inner Context Factors**		
Structure	Structural factors in the clinic (e.g., space, staffing, workflows) that provide an infrastructure to complete program implementation steps.	*Because we were already doing genetic telemedicine we’ve already got it set up. We do have an extra room, space, so we haven’t had any significant issues… As long as we’ve got someone that keeps the room going and makes sure we don’t double book somebody we’re good. (Clinic 3)* *So we were already doing the screening, so that was fairly easy in that we have it electronic where it uploads automatically into the EMR. So that was in place. And then in terms of the referrals, that just goes to our referral coordinator, and is fairly straightforward. (Clinic 1)* *I think maybe one of the reasons why it’s gone so well is that they’ve had the time to – to prioritize it whereas I think if you have bigger, busier practices, it still might be a priority, but so are many other things and if you just don’t have the time to figure out how to do scheduling to figure out how to reschedule someone if they didn’t make it there on their day…that timing probably is a large factor. (Telehealth Team)*
Change Readiness	Clinic and staff commitment, preparedness and ability to take on telehealth program.	*It really wasn’t an issue. We just talked about it being rolled out, and they got it started, and I think everyone was happy to be able to provide the* service. (*Clinic 1*) *Well, the nursing staff definitely didn’t… I mean, it was the OB providers that wanted this, but probably didn’t do enough or we didn’t do enough to kind of champion it to get people comfortable with it. And it didn’t feel like there was room to get in to work with them to make them comfortable. (Telehealth Team)* *There are some groups that really know nothing about this and really don’t want to know anything about it. Then there were others that knew nothing about it but were really receptive to learning about it. (Telehealth Team)*
Culture ^a^	Clinic norms, values, and assumptions regarding change and meeting the maternal mental health and substance abuse needs in their patients.	*She was the one person in that whole entire clinic that got it and thought it was an important service. So that was their culture, “We don’t really know what this is and we don’t really think it’s important.“ (Telehealth Team)* *As soon as you have a pregnant woman that is even just a little bit complicated, they don’t want them in their practice and they send them to [the academic medical center]. And a 100% of our patients are complicated. And we didn’t understand that for a long time. We were like, “Why do you keep sending these poor people here when they could get tele there?“ And so they just did not believe that anybody that was high risk should be in their practice or getting care via tele. (Telehealth Team)* *I think mental health kind of made her [a nurse at the clinic] nervous. She didn’t really buy into it. We went to visit [the site] for a second time to try to revamp them. There was definitely some uncomfortableness she had with mental health. (Telehealth Team)*
Resources	Level of resources/technology dedicated for implementation and on-going operations of telehealth program (e.g., finances, staffing, physical space, time).	*Equipment was the big one. Just everyone knowing how to use it, and so we started having the training for everybody and regular training to make sure everyone knew how to use it. At one point we had two offices and so sometimes they helped one another out about how to use telemedicine. … Making sure it was running, internet connections and all of that was probably the biggest problem initially, but once we got all of that taken care of it wasn’t that difficult. (Clinic 2)* *But I’ll be honest, because we didn’t have one specific person. I’m the clinical supervisor. I help to get the telemedicine…but it was one of our staff that was bringing them back. Again, they were just doing it in between patients. (Clinic 4)* *Our IT was basically needing to connect to their IT and they had no IT person. Or the IT person was – they resourced them out, so they were there only occasionally. Our IT people here were just like, “Well, they don’t have IT. We can’t help you.“ So when we had patients scheduled, that was a big issue…when they had no IT support there. (Telehealth Team)*
Leadership ^a^	Key stakeholder identified and committed to program implementation with accountability.	*If we could have a key person there, that makes the planning so much easier to have somebody who wants this program for the patients and can understand it and is willing to just work through the kinks. That’s really key for planning the scheduling and what’s it going to be like when the patient walks in the door for their tele-appointments. (Telehealth Team)* *But, you know, having that person’s kind of endorsement of us, I think went a really long way with getting a lot of the community and clinic on board. (Telehealth Team)* *They have a nurse… who has I guess championed* [the WRBT program] *more or less from the beginning. She’s taken this program under her wing and helped get everything figured out within the clinic to make it work. (Telehealth Team)*
Teamwork ^a^	Nature and quality of communications and interactions among clinic team members around program implementation.	*It seems like they’re a unified team…I don’t feel like they’re separate entities in the same building or they’re not on the same page and whenever I request [the scheduler] to communicate with [the nurse manager] or vice versa, I see that it happens. I mean, I feel comfortable that it’s happening. (Telehealth Team)* *They had more time to dedicate to get all the staff together to say this is how we’re going to do referrals. This is how it’s all going to work when the patient comes in. I feel like with them, it was just slower to get their groove down and to get the system going. (Telehealth Team)* *There’s a little bit of confusion with the front desk staff. Sometimes you might get one person on the phone who knows how to schedule and they know what you’re talking about or then you might get someone else who’s kind of like, “Oh, I don’t know what this is…” So I feel like they’re not totally on the same page, there’s not great communication happening. (Telehealth Team)*
Attitudes	Beliefs and perceptions of program implementation processes and outcomes.	*It’s definitely an awesome service to have. So we’re grateful for it because our patients don’t need to travel. It was positive. Couldn’t say anything negative about it at all. (Clinic 4)* *And I mean patients are doing much better, happy with the medications and feeling much better, and so when we see them back, I’ve seen a few that saw [a WRBT provider] at two weeks postpartum and then they come back in six weeks and they’re much, much better. So this was very welcome – the patients are loving it and it’s been helpful. (Clinic 3)* *I also feel like their staff and nurses really want to help this population. They really see it as important… they want to pick these people up and get them help. (Telehealth Team)*
**Bridging Factor**		
Telehealth Team-Clinic Partnerships	The relationships built between the telehealth team partners and clinics including the operational style, strategies for support and involvement in implementation of program.	*They were great. I definitely could e-mail…the two physicians or the doctors, I had both of their e-mails. I had [the Telehealth Coordinator’s] phone number and her e-mail. [The telehealth team member] who was in charge, I had all of their phone numbers and they were great… Always responded. (Clinic 4)* *I just think patient educational materials are always good and just making sure we know what’s available through telemedicine, and it’s going to be – word of mouth is a big thing here in this area. And again, when you have a resource available, particularly with those with limited transportation, I think it would be well received. Anything that you can provide us to give to a patient to reassure them that this is an appropriate and accessible resource to use probably will reassure them. (Clinic 5)* *And we weren’t able to ever get in front of them as a group. That never happened. We were just making visits and getting a few key people to get excited about it. I think it’s an important step in that you have a lot of people… getting on the same page and asking questions and understanding what the service is about. (Telehealth Team)*

^a^ Theme endorsed by the telehealth team (Women’s Reproductive Behavioral Health Telemedicine program clinician and staff) only

#### ***Service Demand: Outer Context***

Participants from clinics and the WRBT telemedicine team described that service demand for women’s mental health and substance use disorder treatment was high in rural areas and this acted as a primary facilitator of clinic interest in and willingness to implement the program. All participants described the urgent need for mental health and substance use disorder care in clinic locations as patients lacked access to specialists. While the majority of clinics had mental health screening protocols in place for pregnant and postpartum patients, and obstetrics clinics often also screened for substance use, limited access was available to counselors and treatment as follow-up. Patients needing services therefore had lengthy wait times and often also lacked transportation to specialists. In addition, teams from pediatric practices specifically noted additional complexities with referrals because mothers were technically not their primary patients and best practices for referrals were unclear. Thus, the demand for the telemedicine program was high and valued due to its ability to reduce obstacles to care for their high-risk patients.

#### ***Structure: Inner Context***

Obstetric and pediatric practices brought varied structural characteristics to the WRBT program. Clinics differed in size, number of providers and location, and the telemedicine team described that these factors either promoted or hindered the clinic’s capacity to complete telemedicine steps. Specifically, high patient volumes and limited staffing acted as barriers that deterred telemedicine program adoption. However, many clinics already had pre-existing processes in place for screening women at designated timelines for mental health and substance use challenges that could be adopted to identify potential referrals for the telemedicine program. In addition, clinics with previous telemedicine experience in other areas (e.g., genetics) described comfort with referral processes and management of appointments for the new program.

#### ***Change Readiness: Inner Context***

As described in the Methods section, no formal readiness assessment was conducted with clinics as they joined the program yet participants described that clinic commitment and preparedness to take on the program varied. When readiness was described as high, team members did not hesitate and began making appropriate referrals. In contrast, when readiness was lacking, the WRBT telemedicine team described the need for education regarding the importance of managing mental health and substance use in pregnancy and capitalizing on the infrastructure in place with reminders and additional support to get started.

#### ***Culture: Inner Context***

While the majority of clinics described a clear commitment to caring for women’s well-being that guided their practices, despite these common norms and values across clinics, the telemedicine team uniquely highlighted a practice-wide resistance to addressing mental health and substance use disorder in some clinics. The telemedicine team described that this resistance existed either because clinics did not consider mental health and substance use as problems in their patients or instead because this was not their responsibility. The telemedicine team confirmed that this resistance was a persistent challenge in some clinics as they attempted to implement the program. In fact, one clinic continued to send patients to the academic medical center for mental health care even when the telemedicine program was available on-site in this clinic.

#### ***Resources: Inner Context***

Resources clinics devoted to the telemedicine program varied and impacted program delivery. Critical program resources included dedicated space and staffing and deficits in these areas impacted abilities to carry out the WRBT program. In clinics lacking a designated space for telemedicine visits, participants described the challenge to accommodate visits within current busy schedules. Likewise, having a dedicated staff member to oversee the telemedicine program appeared to facilitate program productivity. A specific need was highlighted for information technology (IT) staff to help troubleshoot technology or connection issues and when this was lacking on-site, this was frustrating for clinic and telemedicine staff. The WRBT telemedicine team also perceived that some clinics had or made time to prioritize the program and integrate it into existing workflow, but others were overwhelmed with competing demands and lacked resources to facilitate efficient implementation of the program. A recommendation for improving feasibility of the program integration and referral process included streamlining the registration process so patients could complete paperwork during the initial in-person clinic visit.

#### ***Leadership: Inner Context***

Only the WRBT telemedicine team described leadership within the clinic as key to program implementation. They believed that program implementation went smoothly when one enthusiastic leader emerged naturally and helped disseminate program details, engage others in the practice and facilitate completion of planning, set-up and scheduling steps. In contrast, those clinics that lacked a solid WRBT program leader tended to lack productivity in scheduling and completing visits or were on a slower overall timeline for program implementation. In some cases, even when an enthusiastic clinician was clearly identified, this did not result in successful implementation if follow-through or detailed planning to fit the program into clinic workflow was lacking.

#### ***Teamwork: Inner Context***

Teamwork among clinic providers and staff was endorsed as important but just from the perspective of the WRBT telemedicine team. They reported that teamwork was often lacking in clinics that struggled with implementation. Specifically, when clinic teams were well coordinated around the technology and program workflow, they functioned better. Strategies that appeared to build teamwork included taking time to bring staff together to complete training, ensuring all team members were knowledgeable about program delivery steps and troubleshooting missed steps and challenges encountered.

#### ***Attitudes: Inner Context***

As telemedicine programs were implemented, positive attitudes about the program in team members appeared to play a role in promoting the program. This was evident most prominently when clinic team members described the program as a valuable resource that met their patients’ needs due to their deep commitment to supporting mental health and substance use challenges. Positive attitudes were also evident when clinic teams highlighted the convenience and feasibility of the program. In addition, a pediatrics office disclosed that they while were initially concerned about ongoing responsibility for mothers who were referred to the program, with an increased understanding of program logistics and understanding that their primary responsibility was to identify women and submit referrals, their concerns diminished.

Clinic and WRBT telemedicine team participants also reported that patients were uniformly satisfied with the program and appreciated the ability to acquire appointments quickly and conveniently. A few clinics described positive attitudes about the program after observing the improvement in patient outcomes when women received telemedicine services. These improvements included clinical outcomes (depression, substance use) as well as overall well-being. One pediatric clinic specifically explained the positive effects of mental health treatment for mothers and its impact on interactions with their infants.

#### ***Telemedicine Team-Clinic Partnership: Bridging Factor***

The partnerships established between the WRBT telemedicine team and clinics varied with some practices working closely with the telemedicine team in a fluid manner and others never establishing trusted relationships. The telemedicine team described that it was critical to build relationships with clinic stakeholders to facilitate understanding of the service and build enthusiasm for the program and its potential benefits. The telemedicine team also outlined the ideal plan for connecting with the full clinic team involved in program delivery but often expressed challenges in completing these steps due to scheduling concerns along with their own staffing shortages to travel and support on-site education. Education was centered on enhancing clinic understanding of workflows to screen, refer and schedule patients. Some clinics described the need for reminders and patient-facing materials.

When strong partnerships were built, clinics described positive communication practices and that it was easy to contact the telemedicine team and access resources from them. The telemedicine team appreciated a flexible approach during ongoing program operations. However, they also described that they did not connect with all clinics in this way. They struggled in developing productive implementation processes in some clinics due to the lack of time and resources at these sites.

## Discussion

To our knowledge, this is the first study to describe barriers and facilitators to implementation of a telemedicine service in obstetric and pediatric clinics for the treatment of PMADs and PSUDs in pregnant and postpartum women. The findings mirror a number of the barriers and facilitators identified in the delivery of telemedicine care in general and in community health centers, highlighting cost, technology, staffing and workflow issues [30, 31]. However, the current study uses an implementation science framework to offer insight into additional contextual and bridging factors and focused on a unique telemedicine service for pregnant women in rural settings for PMAD and PSUD care. Of interest, barriers were common across clinics and across care for PMAD and PSUD, regardless of the amount of time in the program and referral completion rates.

This study demonstrates that the primary program facilitator was demand for services, which was driven by a lack of available or accessible maternal mental health and substance use disorder services for pregnant and postpartum women. Other key facilitators, consistent with existing literature, included the need for infrastructure to support the service including dedicated space, equipment, IT support and staff to facilitate patient scheduling and visits [30, 31]. While different models of program leadership and staffing for the program existed, clinic providers that recognized the importance of addressing maternal mental health and substance use issues were well poised to adopt the telemedicine services quickly and willing to overcome logistical hurdles and other implementation challenges. Telemedicine services appear to be sustained by creating an easy workflow for providers, staff and patients to access services as well as recognizing the benefits that patients received from treatment. Services were further supported by establishing positive reciprocal relationships and communication with the telemedicine team and the clinic staff members who helped facilitate the service. These relationships were not possible in clinics where the service was a low priority and staff turnover was high.

Study results may have implications for potential implementation strategies such as marketing, onboarding and monitoring approaches to support clinics engaging in a telemedicine program for PMADs and PSUDs [32–34]. For example, because service demand was identified as an important driver for program implementation and because clinics highlighted a strong commitment to caring for women’s needs, marketing strategies could centrally emphasize these program benefits and share examples of program-related clinical outcomes. Also, as multiple practical factors were described as influential to implementation such as the structure of the clinic, staffing models and resources available to devote to the program and because these factors varied in clinics, it may be beneficial to administer a needs assessment in the planning stage to identify and address potential clinic gaps and evaluate program feasibility. Finally, as results showed that readiness and teamwork differed across clinics, more research is needed to consider the potential benefits of systematic approaches to prepare clinic teams, identify program champions and trouble-shoot practical challenges to build sustainable programs. Notably only telemedicine team participants emphasized themes of leadership, culture and teamwork as key to successful program implementation. Therefore, it may be beneficial to proactively emphasize these facilitators in program training and planning steps.

### Strengths and limitations

While the strengths of this study included the use of qualitative methods guided by a validated implementation science framework to allow an in-depth exploration of implementation experiences, there are limitations to consider. The study was modest in size with 6 practices and only 1–6 participants per clinic. Even though we used rigorous interviewing techniques and selected participants based on their role in the telemedicine program, this may not reflect the full range of experiences and perceptions at sites as a whole. Also, this study captured clinics at one timepoint and therefore does not capture the dynamics of implementation over time. We chose to conduct online discussion groups with clinics for convenience and this mode may have impacted the level of rapport we were able to build with participants. Finally, as the telemedicine programs launched the WRBT program at different timepoints, this may have impacted participant recall of important start-up implementation processes. Specifically, participants may have had trouble remembering early planning meetings and barriers and facilitators of that process; therefore future prospective methods may supplement our findings by assessing barriers and facilitators over the course of program implementation.

## Conclusion

Access to maternal mental health and substance use disorder treatment for pregnant and postpartum women is paramount in reducing the alarmingly high rates of PMADs and PSUDs and the associated morbidity and mortality associated with these untreated conditions. The use of telemedicine to achieve this end is feasible, acceptable to women and effective in reducing mental health symptoms and substance use [35, 36, 37]. However, there are barriers to implementation of telemedicine services in practice. This study demonstrates and describes these barriers and also highlights facilitators that can strengthen program implementation success. Capitalizing on clinics’ strong commitment to caring for women’s needs, addressing barriers to mental health and substance use disorder and navigating practical resource and IT needs will facilitate program success. Future longitudinal research to better understand the dynamics of telemedicine delivery experiences over time will add to a better understanding of needed implementation strategies to enhance the reach and impact of these programs.

## Electronic Supplementary Material

Below is the link to the electronic supplementary material.

**Supplementary Material 1:** Peer review report.

## Data Availability

The datasets generated and/or analysed during the current study are not publicly available due to confidentiality concerns but de-identified data are available from the corresponding author on reasonable request. The peer review history is available as Supplementary file 1.

## Abbreviations

EPIS: Exploration, Preparation, Implementation, Sustainment
MUSC: Medical University of South Carolina
PMAD: Perinatal Mood and Anxiety Disorders
PSUD: Perinatal Substance Use Disorders
WRBT: Women’s Reproductive Behavioral Health Telemedicine
