# Supplementary material for: Implementation of a women’s reproductive behavioral health telemedicine program: a qualitative study of barriers and facilitators in obstetric and pediatric clinics
Source: BMC Pregnancy Childbirth. 2023 Mar 11;23:167. doi: 10.1186/s12884-023-05463-2 (PMC10007723; doi:10.1186/s12884-023-05463-2)
Supplement: Supplementary file 1 — Supplementary Material 1: Peer review report. [file 12884_2023_5463_MOESM1_ESM.docx]

# Version 1

## Reviewer 1:

The identification and treatment of behavioral health and substance use-related conditions during pregnancy and postpartum is incredibly important. Understanding how we can do this better is an important contribution to the literature. However, in reading through the article, there are several key pieces of information missing (e.g., description of the intervention and relating study findings to existing research). Further, the barriers and facilitators described are largely already known. I'm left wondering if there are any findings that are unique to the PMAD/PSUD space that go beyond what is already reflected to date in telehealth-related implementation barrier/facilitator papers. If so, I would be more deliberate about calling this out.

Background: There is no information provided about the telehealth program (how are people identified/screened for participation, what does the program entail, what types of treatment are provided and how is it provided (especially for SUD which can require in person support if receiving medication assisted treatment)). Also, little information is provided about the OB and peds clinics participating in the study and their connection with the "telehealth team".

Methods: More description is needed about what the online focus groups entail (how were they conducted? Was is via video, did people chat their responses, etc)? Also, What does the "telehealth team" from the academic medical center refer to? Would remove the quoted phrase and provide more description about who these people are. And when it says that the telehealth team completed focus groups, it's difficult to determine if they participated in a focus group or if they facilitated.

Results: The Methods and Results sections are confusing because it is not clear as to whether only focus groups were conducted or if a mixture of focus groups and interviews were utilized (the 1st para in Results makes no mention of interviews, but interviews are referenced in the Methods section above).

Consider providing a range for the number of participants in each focus group (N=18 seems small for 6 focus groups, so should note that some groups only had a couple of people since this is an important limitation).

It's also noted on page 6 that the "telehealth encounters and status in the previous 3 months varied by clinic (0-191)". I'm curious about the site with 0 encounters - was that unusual and how does the 0 encounters relate to the barriers and facilitators identified by this site (e.g., did they experience a major barrier that did not allow them to delver any telehealth in that time period)?

Also, I am wondering if there were any differences uncovered related to the implementation of telehealth for PMAD vs PSUD. While both are considered behavioral health conditions, I imagine that each comes with unique telehealth-related challenges.

Discussion: The authors state that this is the first known study to identify barriers and facilitators to perinatal behavioral health telehealth delivery; however, there is vast literature about the implementation of telehealth in other contexts (especially due to the increase in telehealth use as a result of the COVID-19 pandemic). Instead of rephrasing the Results in the Discussion section, the Discussion should include information about known facilitators and barriers to telehealth implementation and how this study's findings are similar to or different from existing research.

Limitations: Qualitative methods aren't typically meant to be generalizable, so I would not include this as a limitation. A potential limitation would be that you only heard from a couple of staff within some sites which may not reflect the experiences and opinions of the site as a whole.

Minor - some grammatical errors to address (lack of commas where needed, word program used twice in a row on bottom of page 4 and top of page 5_. Also, consider changing "substance abuse" to substance use disorder. If invited for a revise and resubmit, some of the sentences are a bit long or awkward - consider breaking up some of the longer sentences into two. Both "telehealth" and "telemedicine" are used throughout - not sure if this is intentional, but might make sense to choose only one term.

## Reviewer 2:

PEER REVIEWER ASSESSMENTS:

OBJECTIVE - Full research articles: is there a clear objective that addresses a testable research question(s) (brief or other article types: is there a clear objective)?
Yes - there is a clear objective

DESIGN - Is the current approach (including controls and analysis protocols) appropriate for the objective?
Yes - the approach is appropriate

EXECUTION - Are the experiments and analyses performed with technical rigor to allow confidence in the results?
Yes - experiments and analyses were performed appropriately

STATISTICS - Is the use of statistics in the manuscript appropriate?
N/A - there are no statistics in this study

INTERPRETATION - Is the current interpretation/discussion of the results reasonable and not overstated?
Yes - the author's interpretation is reasonable

OVERALL MANUSCRIPT POTENTIAL - Is the current version of this work technically sound? If not, can revisions be made to make the work technically sound?
Yes - current version is technically sound

PEER REVIEWER COMMENTS:

GENERAL COMMENTS:
The objective of this study: to characterise barriers and facilitators to implementing mental health and substance use disorder telemedicine program, is clearly stated and of research value. The design is a cross-sectional study, using qualitative methods (FGDs and interviews) to examine the program.
This is a well-written paper, with the research aim clearly stated and a study that has been appropriately designed and well implemented. Although the sample size was small (6 practices included), the study generated adequate data to address the research aim. The use of the EPIS framework successfully supported the interpretation of the data.

REQUESTED REVISIONS:
I notice in table 2 that the clinics introduced the telemedicine programme at different times (ranging from 2016 - 2019). This bears mentioning in the body of the text, and in the limitations too. I would like the authors to consider the implications that this will have on participants ability to recall information of relevance to the study.
ADDITIONAL REQUESTS/SUGGESTIONS:
The location of the study needs to be mentioned earlier in the study and in the abstract.

The first sentence of the abstract could be made much clearer.

Pg 8 line 24: 'assuring' should be 'ensuring'

## Reviewer 3:

PEER REVIEWER ASSESSMENTS:

OBJECTIVE - Full research articles: is there a clear objective that addresses a testable research question(s) (brief or other article types: is there a clear objective)?
Yes - there is a clear objective

DESIGN - Is the current approach (including controls and analysis protocols) appropriate for the objective?
Yes - the approach is appropriate

EXECUTION - Are the experiments and analyses performed with technical rigor to allow confidence in the results?
Yes - experiments and analyses were performed appropriately

STATISTICS - Is the use of statistics in the manuscript appropriate?
N/A - there are no statistics in this study

INTERPRETATION - Is the current interpretation/discussion of the results reasonable and not overstated?
Yes - the author's interpretation is reasonable

OVERALL MANUSCRIPT POTENTIAL - Is the current version of this work technically sound? If not, can revisions be made to make the work technically sound?
Yes - current version is technically sound

PEER REVIEWER COMMENTS:

GENERAL COMMENTS:
In this qualitative study, the authors aimed to characterize barriers and facilitators to implementation of a telemedicine program for treatment of PMADs and PSUDs in an outpatient community setting. The authors executed the study well and reported their findings using the COREQ checklist which helps in ensuring explicit and comprehensive reporting of qualitative studies in a way that improves the credibility, rigor, and comprehensiveness of results. Well done!

REQUESTED REVISIONS:
On page 6, under ""Participant characteristics"", the authors stated that they conducted 6 online focus groups and two in-person groups. I feel they need to expand on the two in-person focus groups as it is unclear whether:
1. Each of the focus group has 4 participants or two
2. whether there were two focus groups held at different times (with the same 4 respondents).

There would also be need to highlight why the authors chose to do online focus groups and perhaps state the pros and cons of doing this in the ""Strengths and Limitations"" section.

## Authors’ response:

My colleagues and I would like to thank the reviewers for their recent critiques of our manuscript entitled "Implementation of a Women’s Reproductive Behavioral Health Telemedicine Program: A Qualitative Study of Barriers and Facilitators". This manuscript is under consideration for BMC Women’s Health (Manuscript # BMWH-D-21-00453). We appreciate the reviewers’ comments on the strengths of the paper and their excellent suggestions to improve the manuscript. We list each comment below and follow with a description about how we have addressed each critique to strengthen the description of the telemedicine intervention and the reporting of the results. We have also provided a more detailed interpretation of the findings within the existing telemedicine literature. We appreciate this opportunity to revise and resubmit this improved manuscript.

Editor Comments:

The reviewers considered the topic of the manuscript relevant and raised several issues that I agree need to be clarified. The article needs a revision of the writing to correct minor errors and to provide more details related to the methods as detailed in the reviews. The real limitations of the study need to be properly discussed.

Response: Thank you for the opportunity to strengthen this manuscript by revising minor errors in the writing and providing more details about the methods and limitations. Details are provided below regarding how we addressed each critique.

Reviewer #1:

Background Comment: There is no information provided about the telehealth program (how are people identified/screened for participation, what does the program entail, what types of treatment are provided and how is it provided (especially for SUD which can require in person support if receiving medication assisted treatment)).

Response: We agree that the manuscript lacked adequate description of the Women’s Reproductive Behavioral Health Telemedicine (WRBT) program to allow the reader to understand the workflows and relationships between the community clinics and the WRBT clinicians and staff. We have therefore expanded our description of the program in the methods section as follows:

The WRBT was developed at MUSC in 2016 to offer mental health and substance use disorder telemedicine services to community obstetric and pediatric practices. The WRBT Medical Director at MUSC established relationships with clinics through outreach in South Carolina. Protocols were developed for setting up programs in community clinics to deliver mental health and substance use disorder telemedicine services to community clinics’ patients. Clinics employed their own strategies for identifying and screening patients through their clinical care process. Referral processes were also set up between the clinics and MUSC to schedule and complete appointments. All telemedicine visits were conducted on-site at the community clinic. All patients were initially evaluated by a psychiatrist; a treatment plan was created in collaboration with the patients with evidence-based therapy such as Cognitive Behavioral Therapy or Interpersonal Psychotherapy and medication management if appropriate. In accordance with the Ryan Haight Act, patients requiring a prescription for a controlled substance such as buprenorphine were seen in person first per federal and state requirements for prescribing controlled substances via telemedicine.

Background Comment: Also, little information is provided about the OB and peds clinics participating in the study and their connection with the "telehealth team".

Response: We provide details about each clinic in Table 2, including clinic type, number of providers, number of patients seen annually, clinic location, WRBT program launch date and referral numbers, and insurance status and race of patients served. We now expand our description of the clinics in the text of the methods section and in the results section to provide more context. Furthermore, we have added more detail about the interface between the WRBT telemedicine program staff and clinicians with the community clinics in the methods section. Finally, as described below, we have expanded the term “telehealth team” and now refer to the WRBT program clinicians and staff as the “WRBT telemedicine team” after defining this term on page 6. We also use a new footnote at the end of Table 3 to define this term.

Methods Comment: More description is needed about what the online focus groups entail (how were they conducted? Was it via video, did people chat their responses, etc)?

Response: We have provided additional detail about the online focus groups and interviews on page 6. One investigator (KS) served as the moderator and conducted each focus group over Zoom using a structured interview guide. A second investigator (EJ) served as a notetaker, making observations about respondent comments and body language and taking field notes. Each site had video and audio capability over Zoom and all discussion took place in real time (i.e., no chat functions were used).

Methods Comment: Also, what does the "telehealth team" from the academic medical center refer to? Would remove the quoted phrase and provide more description about who these people are. And when it says that the telehealth team completed focus groups, it's difficult to determine if they participated in a focus group or if they facilitated.

Response: As described above, we understand that the term “telehealth team” was hard to interpret and have changed this term to WRBT telemedicine team. We have provided a detailed description about what this term means and who these participants were in the participant characteristics section in the manuscript results.

Results Comment: The Methods and Results sections are confusing because it is not clear as to whether only focus groups were conducted or if a mixture of focus groups and interviews were utilized (the 1st para in Results makes no mention of interviews, but interviews are referenced in the Methods section above). Consider providing a range for the number of participants in each focus group (N=18 seems small for 6 focus groups, so should note that some groups only had a couple of people since this is an important limitation).

Response: We agree this was confusing and have modified the first paragraph of the results to clearly describe the number of focus groups and interviews on page 7. We also now provide a range (number of participants) in the results section and refer to Table 2 where the number of participants per clinic is presented. Finally, we discuss this as an important limitation in the discussion section on page 13.

Results Comment: It's also noted on page 6 that the "telehealth encounters and status in the previous 3 months varied by clinic (0-191)". I'm curious about the site with 0 encounters - was that unusual and how does the 0 encounters relate to the barriers and facilitators identified by this site (e.g., did they experience a major barrier that did not allow them to deliver any telehealth in that time period)?

Response: There were no unique themes identified in the two clinics that did not complete any referrals (Table 2; clinics 2 and 4). Themes were common across sites. We intentionally recruited clinics with varied referral rates into the study to capture a variety of experiences with the telemedicine program but we did not set up the study or the analysis to compare clinics directly and instead examined common themes emerging across clinics. The lack of variability in themes across clinics is now described at the top of page 8 in the results section and further described in the discussion section on page 12.

Results Comment: Also, I am wondering if there were any differences uncovered related to the implementation of telehealth for PMAD vs PSUD. While both are considered behavioral health conditions, I imagine that each comes with unique telehealth-related challenges.

Response: We asked participants about processes and experiences with both PMAD and PSUD telemedicine care and no unique themes were identified. We now describe this in the results section on the top of page 8.

Discussion Comment: The authors state that this is the first known study to identify barriers and facilitators to perinatal behavioral health telehealth delivery; however, there is vast literature about the implementation of telehealth in other contexts (especially due to the increase in telehealth use as a result of the COVID-19 pandemic). Instead of rephrasing the Results in the Discussion section, the Discussion should include information about known facilitators and barriers to telehealth implementation and how this study's findings are similar to or different from existing research.

Response: Thank you for this suggestion to improve the discussion. We have revised the discussion to better describe how our results fit into existing telehealth research and to highlight the results that are unique to PMAD/PSUD. Additional references were added to support the new discussion.

Limitations Comment: Qualitative methods aren't typically meant to be generalizable, so I would not include this as a limitation. A potential limitation would be that you only heard from a couple of staff within some sites which may not reflect the experiences and opinions of the site as a whole.

Response: We have removed the generalizability limitation as recommended and added an additional limitation regarding the small number of participants per site on page 13.

Minor Comments: Some grammatical errors to address (lack of commas where needed, word program used twice in a row on bottom of page 4 and top of page 5). Also, consider changing "substance abuse" to substance use disorder. If invited for a revise and resubmit, some of the sentences are a bit long or awkward - consider breaking up some of the longer sentences into two. Both "telehealth" and "telemedicine" are used throughout - not sure if this is intentional, but might make sense to choose only one term.

Response: We have revised for grammatical errors and changed “substance abuse” to “substance use disorder” throughout the manuscript. We have also changed the word “telehealth” to “telemedicine” throughout the manuscript to better reflect the clinical service delivery in this program. Thank you for these suggestions. Finally, we have edited the full manuscript to break up lengthy sentences.

Reviewer #2 Comments:

This is a well-written paper, with the research aim clearly stated and a study that has been appropriately designed and well implemented. Although the sample size was small (6 practices included), the study generated adequate data to address the research aim. The use of the EPIS framework successfully supported the interpretation of the data.

Response: We appreciate this note regarding the strengths of this study.

I notice in table 2 that the clinics introduced the telemedicine programme at different times (ranging from 2016 - 2019). This bears mentioning in the body of the text, and in the limitations too. I would like the authors to consider the implications that this will have on participants ability to recall information of relevance to the study.

Response: We now describe this point in the results section on page 7 and also note that all respondents were currently involved in the telemedicine program and reflecting on their recent experience. We have also described this as a potential limitation on page 13.

The location of the study needs to be mentioned earlier in the study and in the abstract.

Response: We now note the location in the abstract and methods sections.

The first sentence of the abstract could be made much clearer.

Response: Then first sentence of the abstract has been revised for clarity.

Pg 8 line 24: 'assuring' should be 'ensuring'.

Response: We have made this suggested change.

Reviewer #3 Comments:

The authors executed the study well and reported their findings using the COREQ checklist which helps in ensuring explicit and comprehensive reporting of qualitative studies in a way that improves the credibility, rigor, and comprehensiveness of results. Well done!

Response: Thank you for noting the strength of using the COREQ checklist.

On page 6, under "Participant characteristics", the authors stated that they conducted 6 online focus groups and two in-person groups. I feel they need to expand on the two in-person focus groups as it is unclear whether: 1) Each of the focus group has 4 participants or two and 2) whether there were two focus groups held at different times (with the same 4 respondents).

Response: We agree this was confusing and have now provided more detail about the in-person focus groups on page 7. These two groups were completed with the WRBT telemedicine group at the academic medical center. The same 4 participants (two clinicians, the medical director and a program coordinator for the telemedicine program) were in the two focus groups. Two focus groups were needed to allow adequate time to share their experiences with participating clinics.

There would also be need to highlight why the authors chose to do online focus groups and perhaps state the pros and cons of doing this in the ""Strengths and Limitations"" section."

Response: We have edited the methods section to indicate that we offered online focus groups for convenience for the participants who were busy clinicians (page 6). We also have added a note to the limitations section concerning this modality on page 13.

# Version 2

## Reviewer 1:

Thank you for the opportunity to re-review this article. The authors addressed my previous comments and the current version is much stronger. Very minor - there is a spelling error at the bottom of Table 3 (should say "staff"). One remaining question when reading refers to the "readiness to change" theme starting on page 7. It's not clear whether or not the authors did a formal readiness to change assessment at each site prior to implementation or if this information was gathered qualitatively. If possible to add a bit of clarity, that might be helpful, but the decision to include this or not does not impact my recommendation to approve for publication.

## Reviewer 2:

PEER REVIEWER ASSESSMENTS:

OBJECTIVE - Full research articles: is there a clear objective that addresses one or several testable research questions? (Brief or other article types: is there a clear objective?)
Yes - there is a clear objective

DESIGN - Is the current approach (including controls and analysis protocols) appropriate for the objective?
No - there are minor issues

EXECUTION - Are the experiments and analyses performed with sufficient technical rigor to allow confidence in the results?
No - there are minor issues

STATISTICS - Is the use of statistics in the manuscript appropriate?
N/A - there are no statistics in this study

INTERPRETATION - Is the current interpretation/discussion of the results reasonable and not overstated?
No - there are minor issues

OVERALL MANUSCRIPT POTENTIAL - Has the author addressed your concerns sufficiently for you to now recommend the work as a technically sound contribution? If not, can further revisions be made to make the work technically sound?
Probably - with minor revisions

PEER REVIEWER COMMENTS:

GENERAL COMMENTS: The authors have acknowledged in the limitation section that participant recall may have impacted their reporting of the implementation process. They have done so both in the cover letter and in the limitations section text itself . However I do think the authors need to extend this point a little further, offering some consideration of *how exactly* potential participant difficulties in recall of earlier aspects of the implementation process may have impacted their research findings.

The authors have adequately attended to the more minor edits I suggested in my original review.

The manuscript has been strengthened, in particular in reference to the comments from reviewer #1. However, again, I think the authors need to do more to further clarify the methods and early paragraphs of the results sections of the paper (more detail to follow).

REQUESTED REVISIONS:
As I said above, the authors need to spell out more clearly the impact of recall issues on their results.

I also think the authors need to do more to clear up confusion about their data collection methods (as per reviewer #1 comments). It remains unclear to me whether focus groups or interviews were conducted. This is very important. I am unable to find the information about the number of participants per site in Table 2. Again, I also think that the authors need to draw out the implications of their small sample size further in the discussion/limitations (esp. if participants are <4 in a FGD). It seems likely to me that data from those sites may be considerably thinner.

I am somewhat troubled by the lack of unique themes emerging from the participating clinics, given the intention to include a diversity of clinics in the study. The authors could do more to suggest why this might be. Might the small sample size of the study be behind this? To me it suggests a thinness of data.
ADDITIONAL REQUESTS/SUGGESTIONS:
The word barrier is used twice in the second sentence of the results paragraph in the abstract.

## Reviewer 3:

PEER REVIEWER ASSESSMENTS:

OBJECTIVE - Full research articles: is there a clear objective that addresses one or several testable research questions? (Brief or other article types: is there a clear objective?)
Yes - there is a clear objective

DESIGN - Is the current approach (including controls and analysis protocols) appropriate for the objective?
Yes - the approach is appropriate

EXECUTION - Are the experiments and analyses performed with sufficient technical rigor to allow confidence in the results?
Yes - experiments and analyses were performed appropriately

STATISTICS - Is the use of statistics in the manuscript appropriate?
N/A - there are no statistics in this study

INTERPRETATION - Is the current interpretation/discussion of the results reasonable and not overstated?
Yes - the author's interpretation is reasonable

OVERALL MANUSCRIPT POTENTIAL - Has the author addressed your concerns sufficiently for you to now recommend the work as a technically sound contribution? If not, can further revisions be made to make the work technically sound?
Yes - current version is technically sound

PEER REVIEWER COMMENTS:

GENERAL COMMENTS: The authors have done well to respond to the concerns I raised earlier. Changes have been made in the manuscript in respect of the concerns mentioned.
The revised manuscript has been greatly improved.

REQUESTED REVISIONS:
On page 4, line 33, kindly provide a citation for the Ryan Haight Act.

## Authors’ response:

My colleagues and I have received and addressed the critiques of our recently reviewed resubmission of a manuscript entitled "Implementation of a Women’s Reproductive Behavioral Health Telemedicine Program: A Qualitative Study of Barriers and Facilitators". This manuscript is under consideration for BMC Women’s Health (Manuscript # BMWH-D-21-00453). We list each reviewer comment below and follow with a description about how we have addressed each critique. Thank you.

Editor Comments: Please include the ethics approval for the study in the Ethics approval and consent to participate statement. The statement currently only refers to the waiver of written consent, which is not sufficient.

Response: We have updated the methods section and the Ethics Approval and Consent to Participate section to indicate that this study was approved by the Institutional Review Board at the Medical University of South Carolina.

Reviewer 1 Comments: Very minor - there is a spelling error at the bottom of Table 3 (should say "staff").

Response: Thank you for finding this error. We have fixed the typo.

One remaining question when reading refers to the "readiness to change" theme starting on page 7. It's not clear whether or not the authors did a formal readiness to change assessment at each site prior to implementation or if this information was gathered qualitatively. If possible to add a bit of clarity, that might be helpful, but the decision to include this or not does not impact my recommendation to approve for publication.

Response: No formal readiness assessment was done in this study before launching the telemedicine program. The readiness to change theme was only identified in the qualitative interviews and emerged as a unique theme in the analysis. This is now described for clarity in both the methods section (page 4) when describing the program delivery and in the results section when reporting the readiness theme on the bottom of page 7.

Reviewer 2:

GENERAL COMMENTS: The authors have acknowledged in the limitation section that participant recall may have impacted their reporting of the implementation process. They have done so both in the cover letter and in the limitations section text itself. However I do think the authors need to extend this point a little further, offering some consideration of *how exactly* potential participant difficulties in recall of earlier aspects of the implementation process may have impacted their research findings.

The manuscript has been strengthened, in particular in reference to the comments from reviewer #1. However, again, I think the authors need to do more to further clarify the methods and early paragraphs of the results sections of the paper (more detail to follow).

Response: Please see points below where the reviewer has repeated the above requests.

REQUESTED REVISIONS: As I said above, the authors need to spell out more clearly the impact of recall issues on their results.

We have further described the recall issue concern in the limitations section and emphasized its potential impact on results as well as the future need for longitudinal research in telemedicine to address this concern. We indicate that the primary concern with recall is concerning questions we asked about the start-up period if the program had started several years ago. We state directly: “Specifically, participants may have had trouble remembering early planning meetings and barriers and facilitators of that process; therefore future prospective methods may supplement our findings by assessing barriers and facilitators over the course of program implementation.” One way this concern was decreased was in our recruitment methods as we had practice managers assist in selecting clinicians most knowledgeable about the program to participate. We have updated this recruitment information in the methods section on page 5.

I also think the authors need to do more to clear up confusion about their data collection methods (as per reviewer #1 comments). It remains unclear to me whether focus groups or interviews were conducted. This is very important. I am unable to find the information about the number of participants per site in Table 2. Again, I also think that the authors need to draw out the implications of their small sample size further in the discussion/limitations (esp. if participants are <4 in a FGD). It seems likely to me that data from those sites may be considerably thinner.

Response: Based on ongoing confusion regarding focus groups versus interviews, we have changed the wording from “focus groups” to “small discussion groups” because of the small size of the groups. We had one individual interview at a clinic with a small number of staff and 5 small discussion groups ranging from 2 participants to 6 participants. The sample size and range are described in the text and the sample size for each clinic is listed in the header for each column in Table 2 by clinic. We used rigorous interviewing techniques and identified the key, most knowledgeable clinical staff for each participating clinic to ensure saturation of themes. We offer additional information on identification of key participants in the methods section (page 5) and on the limitation of small numbers in the limitations section on page 12.

I am somewhat troubled by the lack of unique themes emerging from the participating clinics, given the intention to include a diversity of clinics in the study. The authors could do more to suggest why this might be. Might the small sample size of the study be behind this? To me it suggests a thinness of data.

Response: We disagree that there was a “lack of unique themes emerging from the participating clinics”. We used rigorous qualitative data collection and analysis methods and strategies to ensure trustworthiness of our data so have strong assurance that our findings are accurate. We identified 9 unique themes that acted as barriers and facilitators to the implementation of the telemedicine program under study and do not consider this to be a small number of themes. While some of these barriers and facilitators have been identified in previous work in this area as highlighted in our discussion section on the bottom of page 10-top of page 11, our study is unique because we used an implementation science framework to offer insight into additional contextual and bridging factors and focused on a unique telemedicine service for pregnant women in rural settings for PMAD and PSUD care. The small sample size also reflects the small numbers of clinic staff in some of the clinics and our intentional recruitment of those who were most knowledgeable about the program. We have added more detail to the description of recruitment methods (page 5) and to the limitations section to highlight these details (page 12).

ADDITIONAL REQUESTS/SUGGESTIONS:
The word barrier is used twice in the second sentence of the results paragraph in the abstract.

Response: Thank you for this suggestion. The first reference to barriers was changed to challenges in the abstract.

Reviewer 3:

REQUESTED REVISIONS: On page 4, line 33, kindly provide a citation for the Ryan Haight Act.

Response: Thank you for this note. We have added a citation as requested.
